# DISTINGUISHED FACULTY AWARD AND RISING STAR EARLY CAREER FACULTY AWARD PRESENTATIONS AND LECTURES

**DOI:** 10.1093/geroni/igac059.1017

**Published:** 2022-12-20

**Authors:** Kara Dassel

## Abstract

The Distinguished Faculty Award Lecture will feature an address by the 2022 recipient Christine A. Fruhauf, PhD, FGSA, FAGHE, of Colorado State University. The Distinguished Faculty Award recognizes persons whose teaching stands out as exemplary, innovative, of impact, or any combination thereof. The Rising Star Early-Career Faculty Award Lecture will feature an address by 2022 recipient M. Aaron Guest, PhD, MPH, MSW, of Arizona State University. The Rising Star Early-Career Faculty Award acknowledges new faculty whose teaching and leadership stand out as influential and innovative.

